# Supplementary material for: Characterization of HCI-EC-23 a novel estrogen- and progesterone-responsive endometrial cancer cell line
Source: Sci Rep. 2022 Nov 17;12:19731. doi: 10.1038/s41598-022-24211-8 (PMC9672046; doi:10.1038/s41598-022-24211-8)
Supplement: Supplementary file 1 — Supplementary Figures. [file 41598_2022_24211_MOESM1_ESM.pdf]

Supplemental Figures

Characterization of HCI-EC-23 a novel estrogen- and progesterone-responsive endometrial cancer cell line

Craig M. Rush[1], Zannel Blanchard[1], Jacob T. Polaski[1], Kyle S. Osborne[1], Krystle Osby[1], Jeffery M. Vahrenkamp[1], Chieh-Hsiang Yang[1], David H. Lum[2], Christy R. Hagan[3], Kimberly K. Leslie[4], Miles A. Pufall[5], Kristina W. Thiel[6], and Jason Gertz[1,*]

[1]Department of Oncological Sciences, Huntsman Cancer Institute, University of Utah, Salt Lake City, UT, USA
[2]Preclinical Research Resource, Huntsman Cancer Institute, University of Utah, Salt Lake City, UT, USA
[3]Department of Biochemistry and Molecular Biology, University of Kansas Medical Center, Kansas City, KS, USA
[4]Division of Molecular Medicine, Departments of Internal Medicine and Obstetrics and Gynecology, University of New Mexico Comprehensive Cancer Center, University of New Mexico Health Sciences Center, Albuquerque, NM, USA
[5]Department of Biochemistry and Molecular Biology, Holden Comprehensive Cancer Center, Carver College of Medicine, University of Iowa, Iowa City, IA, USA
[6]Department of Obstetrics and Gynecology, University of Iowa, Iowa City, IA, USA
*Lead contact: jay.gertz@hci.utah.edu

Additional Supplemental Files:
Supplemental Table S1. Differentially expressed genes.
Supplemental File 1. Mutation annotation file (MAF) of HCI-EC-23 filtered variants.

Supplemental Figure S1

a

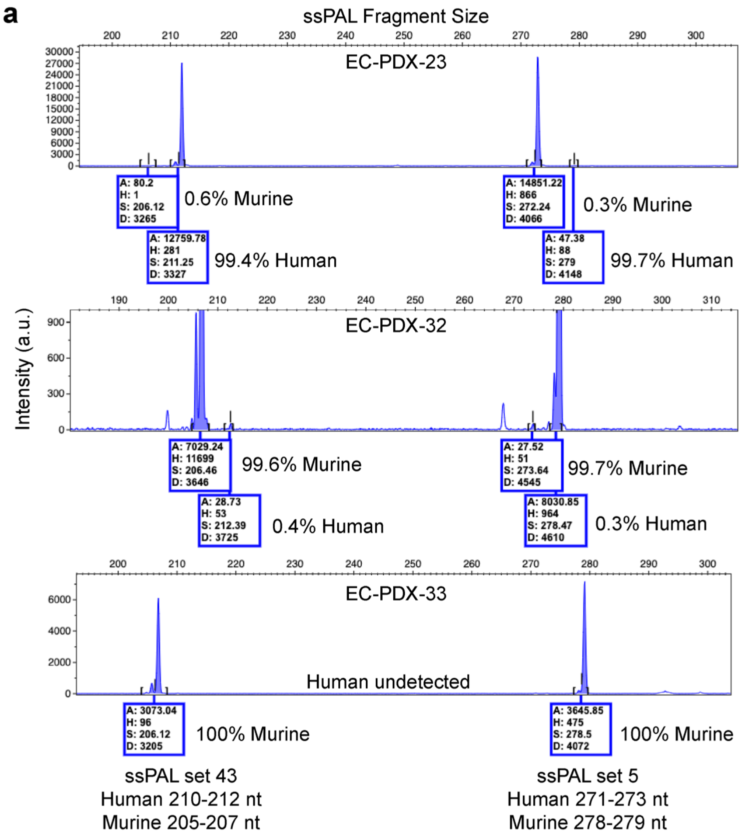

b

IDEXX Species-specific PCR Evaluation	Result
mouse	Negative
rat	Negative
human	Positive
Chinese hamster	Negative
African green monkey	Negative

IDEXX Mycoplasma Evaluation (PCR)	Result
Mycoplasma sp.	Negative

c

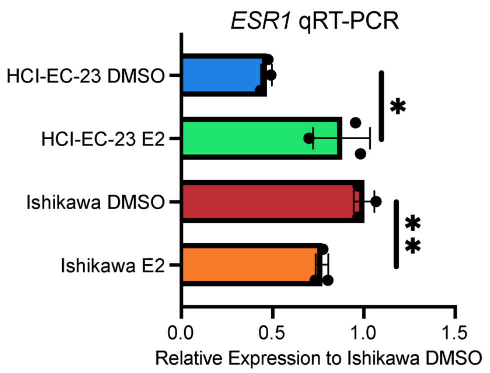

Supplemental Figure S1. Purity of HCI-EC-23 and phenotypic comparison to the Ishikawa cell line (related to Figure 1). (a) Fragment traces from ssPAL of sets 43 (left) and 5 (right) for EC-PDX-23 (top), EC-PDX-32 (middle), and EC-PDX-33 (bottom). Shown are the calculated areas for each murine and human amplicon along with relative percentage. **(b)** Results of commercial testing on HCI-EC-23 by IDEXX for various species (top) and mycoplasma (bottom) contamination. **(c)** Bar plot showing *ESR1* expression as assayed by qRT-PCR in DMSO- or 10 nM E2-treated HCI-EC-23 and Ishikawa cell lines. Data were normalized to internal control *CTCF* and then normalized to DMSO-treated Ishikawa. Error bars represent standard deviation. *p<0.05, **p<0.01.

Supplemental Figure S2

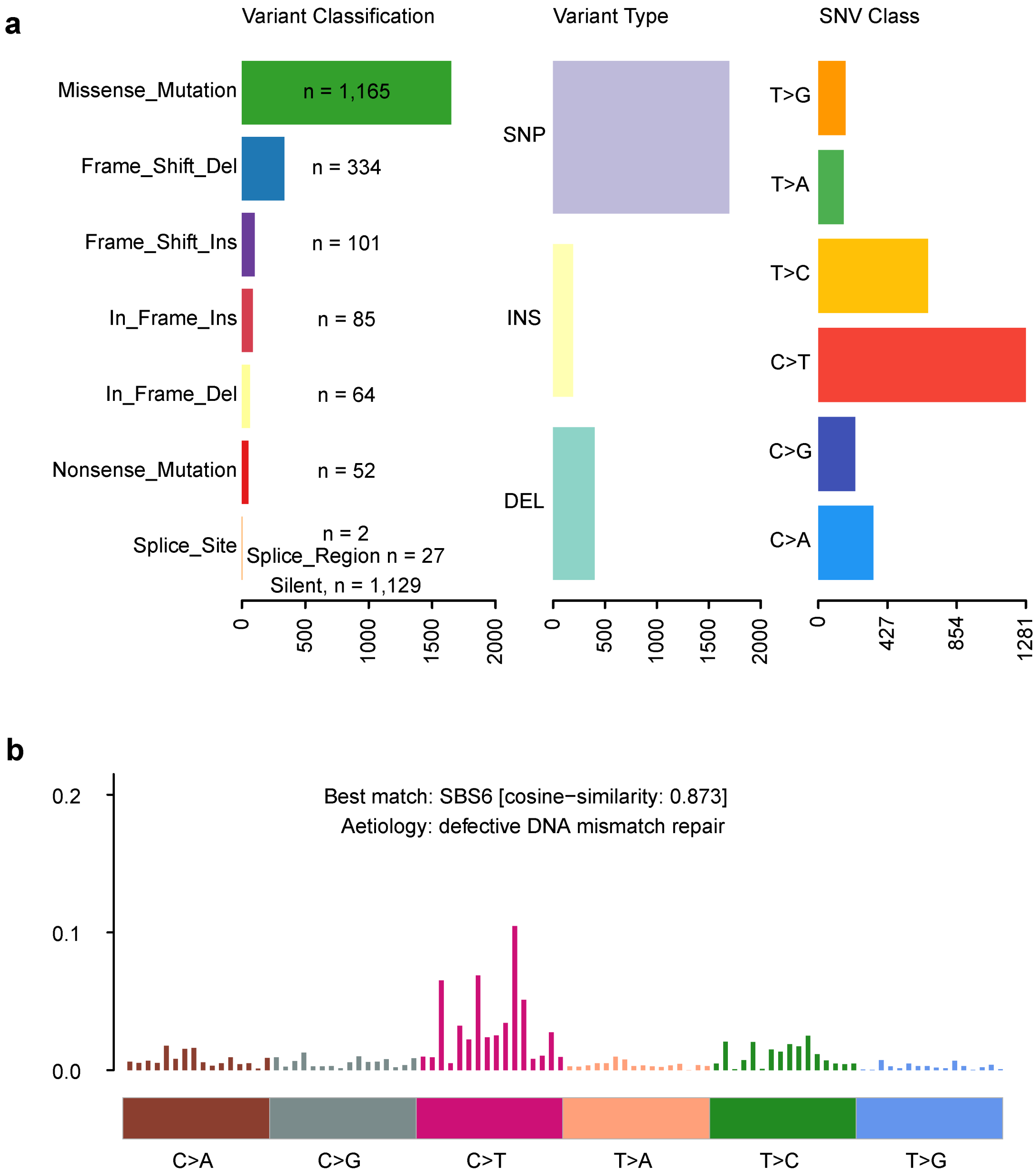

Supplemental Figure S2. Variant class breakdown of HCI-EC-23 mutations and relationship to defective DNA mismatch repair (related to Figure 2). (a) Bar charts displaying the number of variants within specific variant classifications (left), variant type (middle), and base change for SNVs (right). Variant classifications shown are missense, frameshift deletions/insertions, inframe deletions/insertions, nonsense mutations, splice site, splice region, and silent mutations. Variant types are categorized as SNP, insertion, or deletion. **(b)** Mutational signature identified in HCI-EC-23 and its match to SBS6 with a cosine-similarity of 0.873, indicative of DNA mismatch repair defects.

Supplemental Figure S3

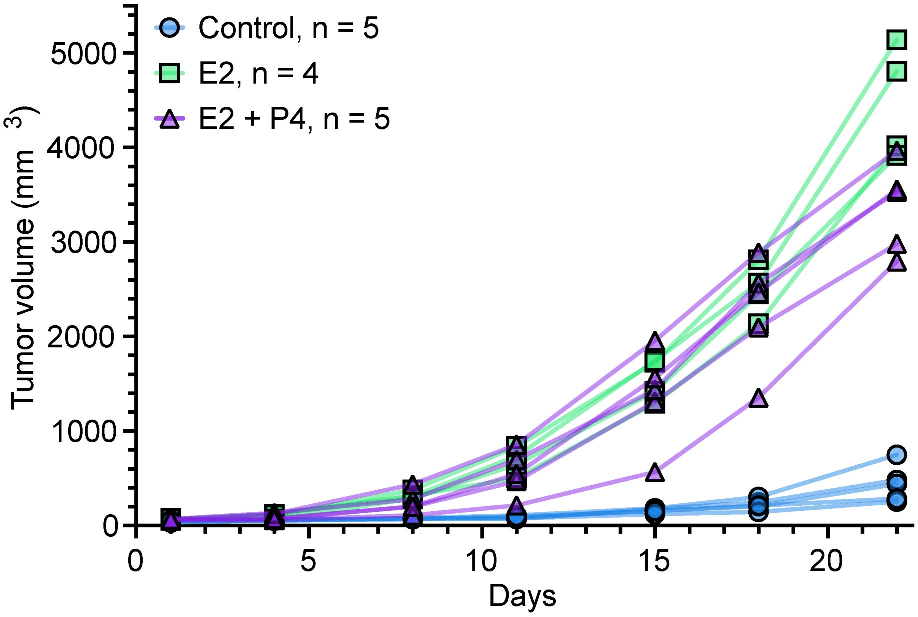

Supplemental Figure S3. Individual xenograft tumor growth (related to Figure 3). Line graph showing individual xenograft tumor growth of HCI-EC-23 xenografts in ovariectomized, immunocompromised mice. 1x10^6 cells were injected into the flank of mice treated with E2 (n=4), E2+P4 (n=5), or control (n=5) pellets. Day 1 represents the first day of measurement and was 10 days post-injection.

Supplemental Figure S4

a

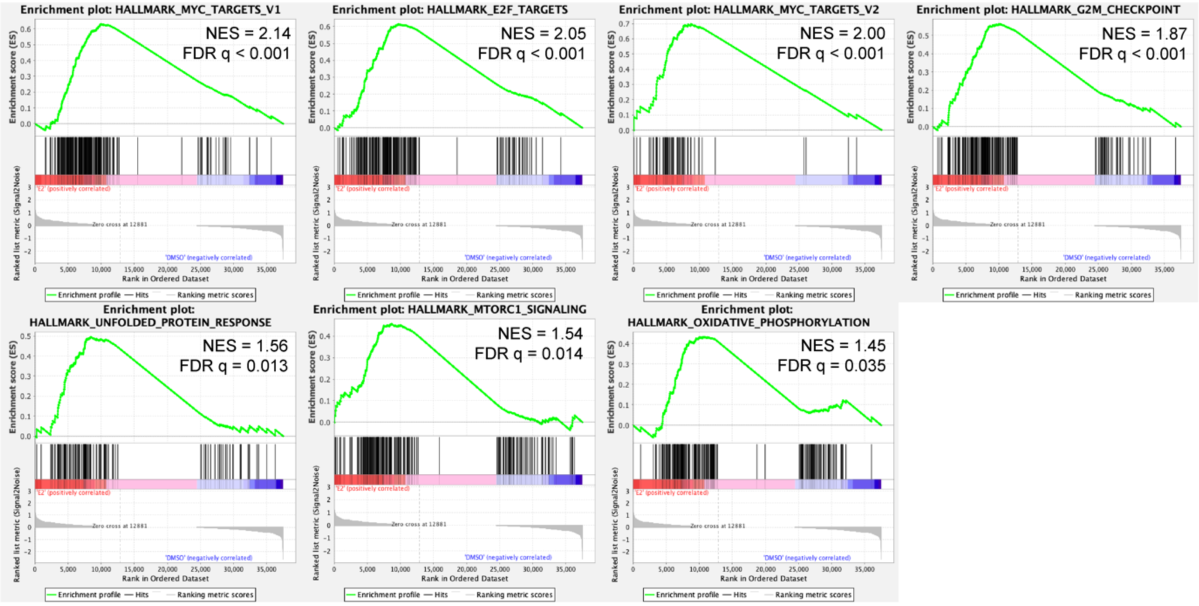

b

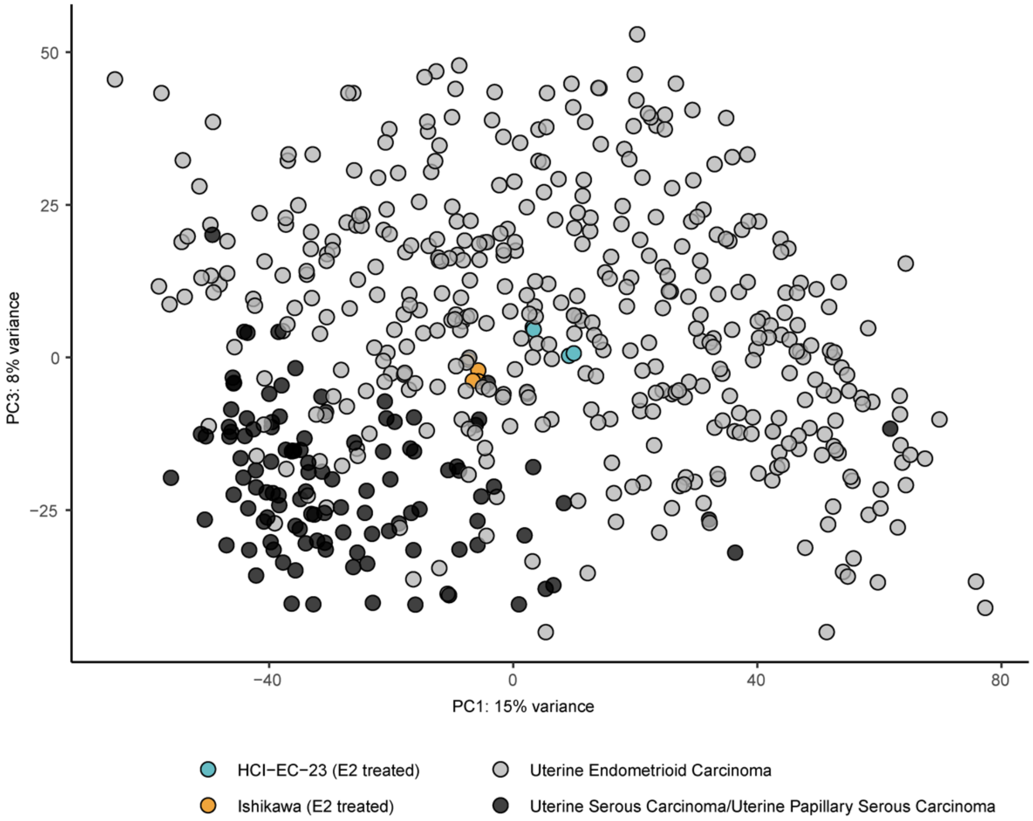

Supplemental Figure S4. Additional enriched pathways in HCI-EC-23 E2-treated RNA-seq differentially expressed genes and comparison of HCI-EC-23 and Ishikawa RNA-seq to TCGA UCEC cohort (related to Figure 4). (a) Gene set enrichment analysis (GSEA) plots for additional hallmark GSEA sets shown in Figure 1B. NES is the normalized enrichment score. FDR q-value is the false discovery rate. **(b)** Principal component analysis (PCA) plot of RNA-seq data for E2 treated HCI-EC-23 (blue), E2 treated Ishikawa (orange), TCGA uterine endometrioid carcinoma (gray), and TCGA uterine serous/papillary serous carcinoma. The serous and papillary serous tumors segregate from the majority of endometrioid tumors. The HCI-EC-23 and Ishikawa cell lines cluster with the endometrioid tumors.

Supplemental Figure S5

a

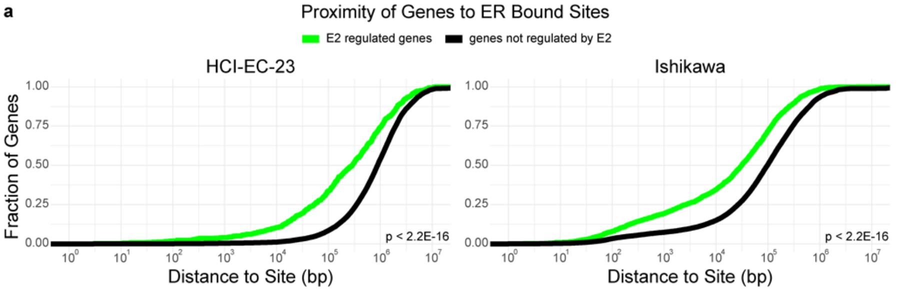

b

Shared ER ChIP-seq Sites

Motif Found	Known or Similar Motif	HCI-EC-23 Estrogen Receptor ChIP E-Value Replicate 1	HCI-EC-23 Estrogen Receptor ChIP E-Value Replicate 2	Ishikawa Estrogen Receptor ChIP E-Value Replicate 1	Ishikawa Estrogen Receptor ChIP E-Value Replicate 2
	ERE (ER)	4.8E-691	3.0E-620	8.6E-572	3.0E-562
	PITX2	5.5E-25	1.1E-18	4.9E-36	n.s.
	ZN770, MAF, ZSC22, SMAD3	1.4E-14	1.1E-14	1.8E-17	2.1E-06

HCI-EC-23-Specific ER ChIP-seq Sites

Motif Found	Known or Similar Motif	E-Value Replicate 1	E-Value Replicate 2
	ERE (ER)	1.2E-242	1.8E-224
	ATF3	2.8E-11	1.2E-15
	ZN770, ZSC22, ZFX, CRX	5.5E-07	4.1E-07

Ishikawa-Specific ER ChIP-seq Sites

Motif Found	Known or Similar Motif	E-Value Replicate 1	E-Value Replicate 2
	ERE (ER)	4.9E-1022	2.1E-905
	AP-1	5.1E-165	6.4E76
	PITX2, ZN264	3.1E-103	5.8E-60

c

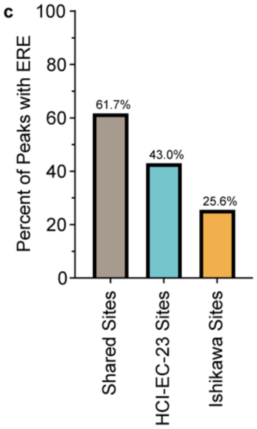

d

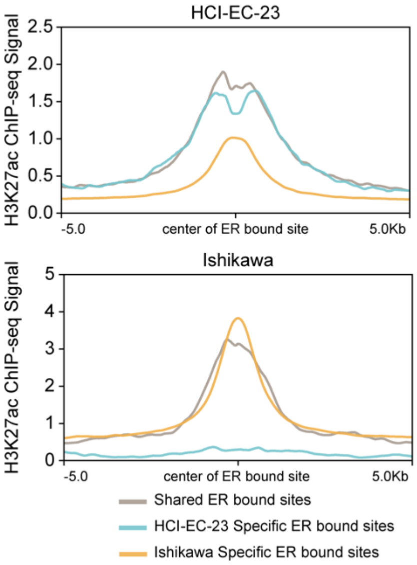

e

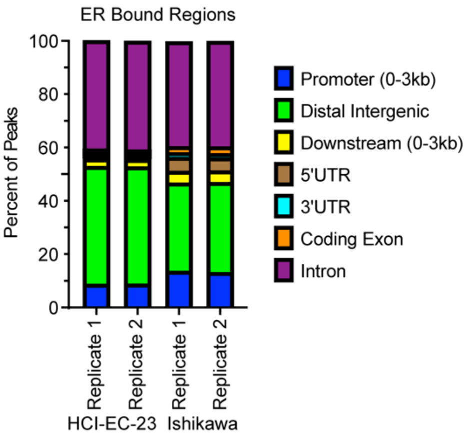

Supplemental Figure S5. Motif analysis and genomic distribution of ER ChIP-seq peaks in HCI-EC-23 and Ishikawa (related to Figure 5). (a) Cumulative distance plots show the fraction of E2 regulated and not regulated genes with a ER bound site within specified distance from their transcription start sites. Shown are distances for E2 regulated and not regulated genes to ER bound sites in HCI-EC-23 (left) or Ishikawa (right). Wilcoxon signed rank test p value is shown. **(b)** Top 3 motifs identified across replicates in shared (top), HCI-EC-23-specific (middle), and Ishikawa-specific (bottom) ER ChIP-seq peaks. Shown are the identified motif logo along with factors with known or similar motifs. E-values for statistical strength of motif within each set is shown by replicate. **(c)** Bar plot showing percentage of peaks harboring an ERE motif for shared (left), HCI-EC-23-specific (middle), and Ishikawa-specific (right) ChIP-seq peaks. **(d)** Profile plots are shown for H3K27ac ChIP-seq signal centered at ER bound sites. Profiles are colored according to category of ER bound site either shared, HCI-EC-23 specific, or Ishikawa specific, as indicated in the legend. Plots represent averaged signal between two ChIP-seq replicates. **(e)** Bar plots show genomic distribution of ER ChIP-seq peaks. Shown are each replicate for HCI-EC-23 and Ishikawa.

Source Data Figure 1a

Raw 800CW channel (Santa Cruz α-ERα sc-543)

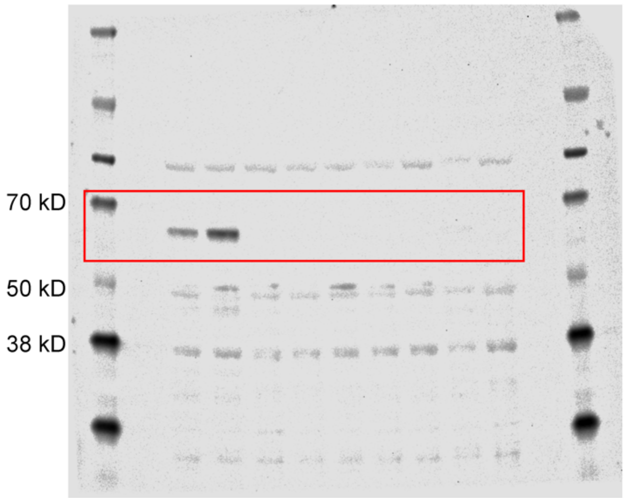

Raw 680RD channel (Santa Cruz α-Actin sc-47778)

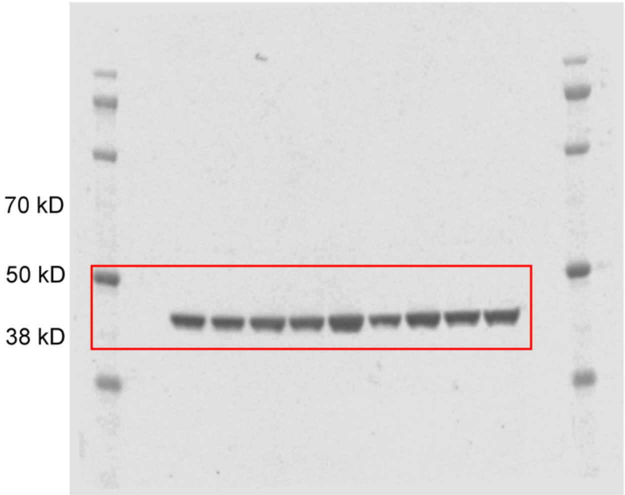

Source Data for Figure 1a. Provided are the raw blot images for the western blots in Figure 1a. Red boxes indicate cropped regions used for Figure 1a.
